# Percutaneous Thrombectomy for Clot-in-Transit With Concomitant Left Ventricular Thrombus in a Patient With Acute Heart Failure Exacerbation: A Case Report

**DOI:** 10.7759/cureus.110842

**Published:** 2026-06-14

**Authors:** Ebram Eskandar, Sachin Joseph, Andres Vargas Estrada

**Affiliations:** 1 Internal Medicine, Florida State University, Tallahassee, USA; 2 Interventional Cardiology, Tallahassee Memorial HealthCare, Tallahassee, USA

**Keywords:** clot-in-transit, left ventricular thrombus, percutaneous mechanical thrombectomy, pulmonary embolism, right ventricle thrombus

## Abstract

Clot-in-transit (CIT) is a life-threatening condition in which a venous thrombus is visualized within the right heart while migrating toward the pulmonary arteries. Its coexistence with severe cardiomyopathy and a left ventricular thrombus confers high morbidity and mortality. We report the case of a woman in her 40s with advanced heart failure with reduced ejection fraction (HFrEF), with a left ventricular ejection fraction (LVEF) of 10%-15%, who presented with acute pulmonary embolism (PE) , a large mobile thrombus traversing the tricuspid valve, and an apical left ventricular thrombus, complicated by cardiogenic shock. Given her hemodynamic instability and high surgical risk, she underwent emergent percutaneous aspiration thrombectomy using the FlowTriever device (Inari Medical, Inc., Irvine, CA), achieving successful clot removal and hemodynamic improvement. This case highlights the role of minimally invasive thrombectomy in selected high-risk patients.

## Introduction

Clot-in-transit (CIT) is an uncommon, life-threatening complication of venous thromboembolism (VTE). It is characterized by a thrombus visible in the right-sided cardiac chambers as it travels toward the pulmonary arterial circulation. In the setting of acute pulmonary embolism (PE), CIT is associated with an increased risk of hemodynamic deterioration and mortality. However, optimal management remains under investigation [1]. Current treatment options include anticoagulation, systemic thrombolysis, surgical embolectomy, and percutaneous aspiration thrombectomy. The choice of therapy is guided by hemodynamic status, bleeding risk, clot burden, and patient-specific comorbidities, as reflected in the 2026 risk stratification framework for PE [1-3]. Recently, percutaneous aspiration thrombectomy has emerged as a minimally invasive option for selected patients with acute PE, yet evidence for its use in patients with concomitant intracardiac thrombi remains limited [4]. Here, we report a case of acute PE with right-sided CIT and a concomitant left ventricular thrombus in a patient with advanced heart failure who was successfully treated with percutaneous aspiration thrombectomy.

## Case presentation

A woman in her late 40s with a significant medical history of heart failure with reduced ejection fraction (HFrEF) secondary to nonischemic cardiomyopathy, under evaluation for a possible infiltrative process, presented to an outside hospital with a two-day history of progressive cough, shortness of breath, and right upper quadrant pain. She initially attributed her symptoms to a possible respiratory infection and was prescribed Augmentin and Tessalon Perles by her primary care physician.

On the morning of admission, she experienced one episode of hemoptysis. Given symptom progression despite treatment, she sought emergency care. At the outside hospital, chest X-ray showed right lower lobe infiltrates suggestive of pneumonia versus pulmonary hemorrhage. CT angiography revealed a large right main pulmonary artery (PA) embolism and left upper lobe PE (Figure 1). Venous duplex ultrasound demonstrated a right popliteal deep venous thrombosis (DVT) (Figure 2). She was started on anticoagulation with a bolus and infusion of unfractionated heparin and was transferred to our facility for further management and potential intervention.

**Figure 1:**
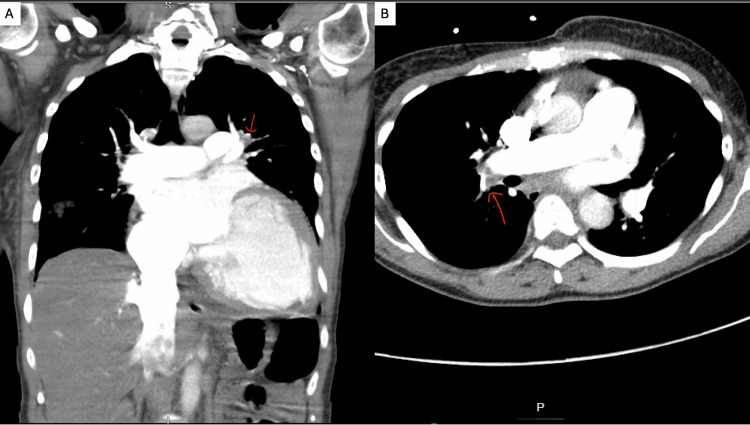
CT pulmonary angiography demonstrating pulmonary embolism (PE). A. Coronal contrast-enhanced CT image showing an intraluminal filling defect within a left upper lobe pulmonary arterial branch. B. Axial CT image demonstrating a right main pulmonary artery filling defect, consistent with acute pulmonary embolism.

**Figure 2:**
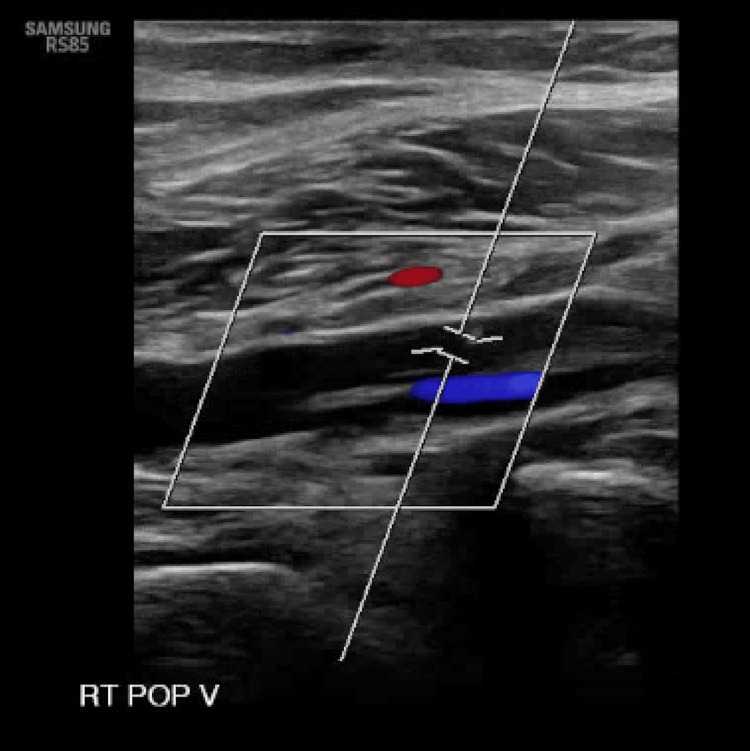
Right lower-extremity venous duplex ultrasound demonstrating DVT. Color Doppler image of the right popliteal vein demonstrating an intraluminal thrombus with reduced or absent venous flow, consistent with right popliteal DVT. DVT: Deep vein thrombosis.

Upon arrival at our institution, the patient was hemodynamically stable, with a blood pressure of 110/80 mmHg, heart rate of 88 bpm, respiratory rate of 20 breaths per minute, and oxygen saturation of 94% on room air. Pertinent laboratory investigations on presentation are summarized in Table 1. Laboratory evaluation was notable for elevated D-dimer, markedly elevated brain natriuretic peptide (BNP), normal high-sensitivity troponin I, and mildly elevated lactate.

**Table 1:** Pertinent laboratory investigations on presentation.

Laboratory test	Patient value	Reference range	Interpretation
D-dimer	11.2 mg/L FEU	<0.50 mg/L FEU	Elevated
High-sensitivity troponin I	12 ng/L	≤49 ng/L; values >49 ng/L exceed the 99th percentile upper reference limit	Within normal range; not elevated
Brain natriuretic peptide (BNP)	1,784 pg/mL	<100 pg/mL	Markedly elevated
Lactate	2.3 mmol/L	0.5-2.0 mmol/L	Mildly elevated

Transthoracic echocardiography (TTE) was performed urgently and revealed a severely dilated left ventricle (left ventricular end-diastolic diameter (LVEDD) 8.3 cm) with severely reduced systolic function (left ventricular ejection fraction (LVEF) 10%-15%), extensive anterior and apical akinesis with global hypokinesis, and a 1.9 × 1.0 cm left ventricular apical thrombus. A large mobile thrombus was seen traversing the tricuspid valve from the right atrium into the right ventricle, consistent with CIT (Figure 3). Additional findings included right ventricular enlargement with reduced systolic function, biatrial enlargement, moderate-to-severe tricuspid regurgitation, severe pulmonary hypertension, an estimated pulmonary artery systolic pressure (PASP) of 64 mmHg, and no evidence of interatrial shunt (Video 1). Compared with baseline TTE findings, there was acute worsening of tricuspid regurgitation and pulmonary pressures. In the cardiac catheterization laboratory, lactate was 2.3 mmol/L, consistent with early normotensive cardiogenic shock in the setting of acute PE, corresponding to the 2026 American Heart Association/American College of Cardiology (AHA/ACC) Acute PE Clinical Category C3-R, with concern for progression toward D2.

**Figure 3:**
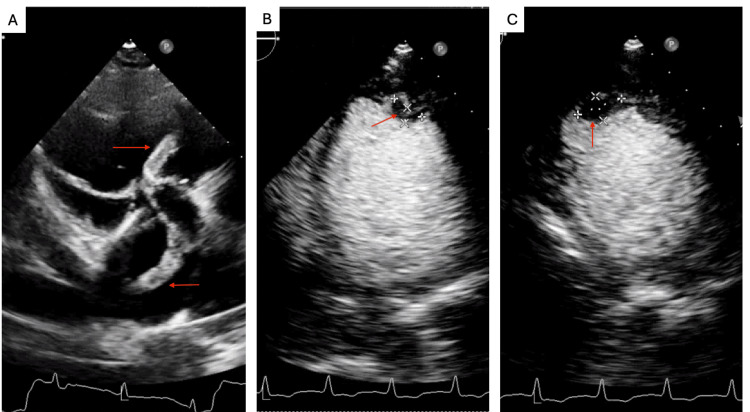
Transthoracic echocardiography (TTE) demonstrating right-sided clot-in-transit and left ventricular apical thrombus. A. Echocardiographic view demonstrating a mobile right-sided intracardiac thrombus prolapsing across the tricuspid valve, indicated by the red arrows. B-C. Contrast-enhanced TTE images demonstrating a left ventricular apical filling defect consistent with thrombus, indicated by the red arrows and measurement calipers.

**Video 1:** Transthoracic echocardiography (TTE) visualization of clot-in-transit (CIT). Real-time TTE demonstrates a large, mobile right-sided intracardiac thrombus prolapsing across the tricuspid valve, consistent with CIT.

For risk stratification, the simplified Pulmonary Embolism Severity Index (sPESI) was 1, indicating elevated mortality risk; the Bova score was 4 points (Stage II), indicating intermediate risk for PE-related complications; and the venous thromboembolism bleeding risk score (VTE-BLEED), a tool used to estimate bleeding risk during anticoagulation in patients with VTE, was 1.5 points, indicating low bleeding risk. In parallel, the 2026 AHA/ACC Acute PE Clinical Categories placed her in a high-severity normotensive category because of right ventricular dysfunction, elevated BNP, severe underlying cardiomyopathy, and concern for impending hemodynamic deterioration [3].

Management and procedure

Given the critical nature of her presentation, including CIT, elevated pulmonary pressures, borderline hemodynamics, acute worsening of tricuspid regurgitation, and severe underlying cardiomyopathy with an LVEF of 10%-15%, emergent percutaneous mechanical aspiration thrombectomy was recommended following multidisciplinary heart-team discussion.

General anesthesia with transesophageal echocardiography (TEE) monitoring was selected despite intermediate-risk PE, as it allowed airway control, procedural stability, and real-time visualization of the mobile right-sided thrombus (Figure 4). Venoarterial extracorporeal membrane oxygenation (VA-ECMO) backup was considered, given the risk of acute decompensation in the setting of severe cardiomyopathy. Right femoral venous access was obtained with a 6-Fr sheath under fluoroscopic guidance. Right heart catheterization using a Swan-Ganz catheter demonstrated elevated filling pressures, including a right atrial pressure of 27 mmHg and PA pressure of 68/37 mmHg, with a mean PA pressure of 46 mmHg, consistent with severe pulmonary hypertension.

**Figure 4:**
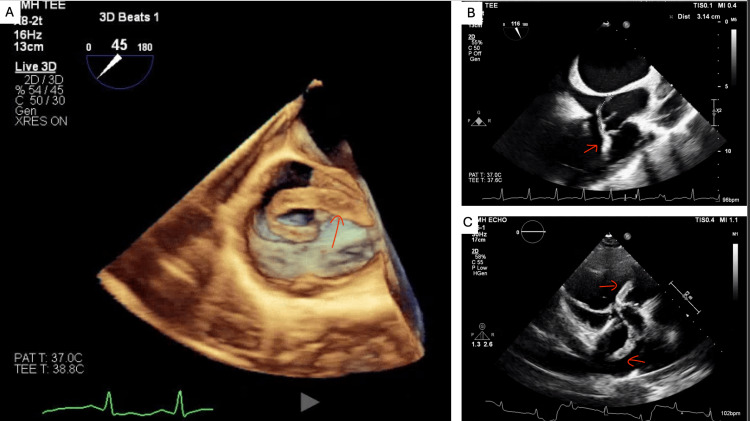
Three-dimensional (3D) and two-dimensional (2D) echocardiographic characterization of right-sided clot-in-transit. A. Three-dimensional transesophageal echocardiographic en face view of the tricuspid valve demonstrating an irregular, mobile echogenic mass at or near the tricuspid valve, indicated by the red arrow. B. Two-dimensional transesophageal echocardiographic view demonstrating a mobile right-sided thrombus, indicated by the red arrow. C. Echocardiographic view demonstrating a mobile right-sided intracardiac echodensity prolapsing across the tricuspid valve, consistent with clot-in-transit, indicated by the red arrows.

Following right heart catheterization, the access site was serially dilated and exchanged over a stiff wire to accommodate a large-bore introducer sheath. Although an AngioVac strategy was considered, including an 18-Fr sheath for the PE component and a 22-Fr sheath for the right atrial clot, the Inari FlowTriever system was chosen because the procedural intent was to perform PE thrombectomy and rapidly address both the right-sided CIT and pulmonary arterial thrombus burden with a single system in the setting of acute illness.

A 24-Fr FlowTriever catheter (Inari Medical, Inc., Irvine, CA) was advanced into the right atrium under fluoroscopic and echocardiographic guidance. The device tip was positioned within the thrombus lodged at the tricuspid valve, and gentle suction was applied to engage the clot material. Five aspiration passes were performed, with successful retrieval of red and white thrombus material. Transesophageal echocardiography (TEE) confirmed progressive reduction in clot size and improved flow across the tricuspid valve (Figure 5).

**Figure 5:**
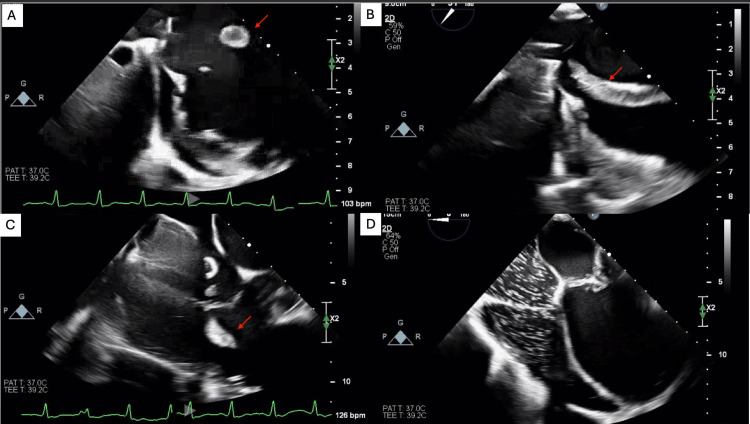
Transesophageal echocardiography (TEE) guidance during aspiration thrombectomy of right-sided clot-in-transit (CIT). A-C. Sequential TEE views demonstrating FlowTriever aspiration thrombectomy of a large, mobile right-sided intracardiac thrombus. Red arrows indicate the mobile echogenic thrombus within the right-sided cardiac chambers. D. Reinfusion of aspirated blood into the right-sided circulation.

After successful removal of the clot-in-transit from the right heart, attention was turned to the PA thrombus burden. Pulmonary angiography was performed via a pigtail catheter to delineate the extent and location of the PE. Bilateral PA access was obtained. In the right PA, five aspiration passes were performed in the right main PA and right interlobar branch, with retrieval of red clot material. In the left PA, four aspiration passes were performed in the left main PA and left interlobar branch, with removal of a significant red clot burden. Repeat pulmonary angiography demonstrated a significant bilateral reduction in thrombus burden, with improved distal pulmonary perfusion (Figure 6).

**Figure 6:**
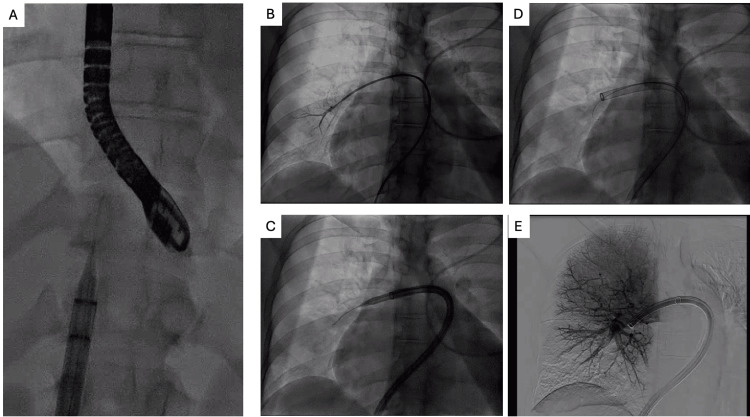
Fluoroscopic stages of percutaneous aspiration thrombectomy. A. Large-bore aspiration catheter positioned within the right heart and pulmonary arterial circulation. B. Pulmonary angiography demonstrating right pulmonary arterial filling defects. C-D. Catheter advancement and repositioning for targeted aspiration thrombectomy. E. Post-thrombectomy angiography demonstrating improved distal pulmonary perfusion.

Repeat intracardiac pressure measurements demonstrated improvement in PASP from 68 mmHg to 59 mmHg and mean PA pressure from 46 mmHg to 41 mmHg. Right atrial pressure remained elevated at 25 mmHg, which was expected immediately after thrombectomy given persistent severe cardiomyopathy and elevated filling pressures.

Overall post-procedure hemodynamic improvement was noted, reflecting reduced right ventricular afterload after removal of the proximal and distal thrombus burden. Notably, the patient’s heart rate had increased from 88 bpm on arrival to 120 bpm before the procedure, suggesting interval hemodynamic deterioration; after the procedure, the heart rate decreased to 110 bpm.

The right femoral venous sheath was left in place for continued venous access and hemodynamic monitoring, and a FlowStasis device was applied at the venous access site.

Post-procedure course

Following mechanical thrombectomy, the patient was managed in the cardiac ICU after procedural intubation and was successfully extubated on post-procedure day 1. She required IV diuresis, temporary milrinone, and vasopressor support for cardiogenic shock and elevated filling pressures, with progressive improvement over the following days. Lactate improved to 1.2 mmol/L by post-procedure day 2. She also received empiric piperacillin-tazobactam for suspected bacterial pneumonia, with improvement in fever and leukocytosis. By post-procedure days 3-4, vasopressors were discontinued, and she maintained adequate blood pressure on oral midodrine. At discharge on post-procedure day 5, she was hemodynamically stable, tolerating oral intake, and ambulating independently.

Anticoagulation was transitioned from unfractionated heparin to apixaban 10 mg twice daily for the first week, followed by 5 mg twice daily for at least 6 months. The left ventricular apical thrombus was managed medically with systemic anticoagulation rather than procedural intervention. She was referred to the Advanced Heart Failure Clinic for guideline-directed medical therapy (GDMT) optimization, advanced therapies evaluation, and implantable cardioverter-defibrillator (ICD) evaluation, as well as to hematology for thrombophilia evaluation given the extensive VTE burden with unclear provoking factors. Repeat TTE was planned at 3 months to reassess the left ventricular thrombus, PA systolic pressure, and right ventricular size and function.

## Discussion

The combination of CIT, bilateral PE, severe HFrEF with an LVEF of 10%-15%, and concomitant left ventricular apical thrombus in this case represents a uniquely complex and previously undescribed clinical scenario. The complexity was compounded by acute-on-chronic cardiogenic shock physiology, acute functional tricuspid regurgitation, and the need for simultaneous management of right-sided and left-sided thrombus burden.

CIT adherent to the tricuspid valve represents an uncommon variant of right heart thrombus with unique pathophysiologic implications. Kim KW et al. described a 96-year-old man with submassive PE complicated by CIT adherent to the tricuspid valve who was successfully managed with mechanical thrombectomy using the Inari FlowTriever device [5]. This case demonstrated that percutaneous intervention is feasible in selected high-risk scenarios. When CIT is combined with bilateral PE and hemodynamic compromise, mortality rates may reach 40%-50% without immediate intervention, making this among the most catastrophic presentations of acute PE [1].

Few studies have evaluated optimal treatment strategies for CIT. In a meta-analysis comparing anticoagulation with invasive and noninvasive strategies, Maqsood MH et al. reported a weighted mortality of 35% (95% CI, 21%-49%) with anticoagulation alone and 20% (95% CI, 9%-34%) with catheter-based thrombectomy [1]. Zhang RS et al. conducted the largest comparative study of CIT management strategies to date, including 35 patients, and demonstrated that catheter-based therapy was associated with significantly lower mortality compared with anticoagulation alone (6% vs. 50%; hazard ratio, 0.10) [6]. The FlowTriever for Acute Massive Pulmonary Embolism (FLAME) study demonstrated improved outcomes with mechanical thrombectomy in high-risk PE; however, patients with clot-in-transit were not specifically included, so extrapolation to intracardiac thrombi should be interpreted cautiously [4]. Recent case reports have described successful aspiration thrombectomy for CIT, including a 37-year-old man with bilateral PE treated with the FlowTriever device [7]. However, there is no consensus on the optimal treatment modality for CIT, and the 2026 AHA/ACC guideline acknowledges that high-quality comparative data addressing this population are currently unavailable [3]. Additional data are needed.

A critical diagnostic insight from this case is the recognition of normotensive cardiogenic shock. Despite relatively reassuring vital signs, including a blood pressure of 110/80 mmHg and heart rate of 88 bpm, the patient had evidence of early hemodynamic compromise, including elevated lactate (2.3 mmol/L), BNP of 1,784 pg/mL, right ventricular dysfunction, and markedly elevated filling pressures, including a right atrial pressure of 27 mmHg and PA pressure of 68/37 mmHg. This illustrates that acute PE can produce right ventricular-pulmonary arterial (RV-PA) uncoupling and impending shock despite preserved systemic blood pressure. Because only a minority of patients with PE present with persistent hypotension, obstructive shock, or cardiac arrest, risk stratification of normotensive PE remains essential. In this case, Bova staging and the 2026 AHA/ACC Acute PE Clinical Categories supported escalation to catheter-based therapy in the setting of high-risk features and concern for deterioration [2,3].

The safety profile of the FlowTriever system is supported by the FLAME study, which demonstrated low in-hospital mortality (1.9%) in high-risk PE [4]. However, real-world Manufacturer and User Facility Device Experience (MAUDE) database analyses have identified rare but serious complications, including pulmonary vascular perforation and cardiac perforation/tamponade; Warren BE et al. identified 26 FlowTriever-related mortality events, with pulmonary vascular perforation and right heart injury as the most common causes, although these reports do not provide true incidence rates because they lack a denominator [8]. The risk of distal embolization during aspiration thrombectomy can be mitigated by continuous large-bore aspiration, en bloc clot extraction, and careful catheter manipulation.

In this case, apixaban was chosen over warfarin for management of the left ventricular apical thrombus. The 2022 AHA Scientific Statement on left ventricular thrombus management included an updated meta-analysis of 21 studies involving 3,057 patients and found no significant differences between direct oral anticoagulants (DOACs) and vitamin K antagonists (VKAs) in stroke or systemic embolization, all-cause mortality, thrombus resolution, or bleeding, concluding that DOACs represent a reasonable alternative to VKAs [9]. Apixaban was favored for this patient, given the practical advantages of fixed dosing without the need for regular INR monitoring and the complexity of her medical regimen. However, it should be acknowledged that large randomized trials are lacking, and close outpatient follow-up was planned to confirm thrombus resolution.

Limitations

This report is subject to the inherent limitations of a single case. The observed hemodynamic improvement cannot be definitively attributed to thrombectomy alone, as concurrent medical therapy, diuresis, and milrinone may also have contributed. Long-term follow-up data, including left ventricular thrombus resolution and recurrent VTE, are not yet available. Case reports are subject to selection and publication biases, and the findings cannot be generalized without prospective comparative data.

## Conclusions

CIT in the setting of left ventricular thrombus, acute bilateral pulmonary embolism, and severe HFrEF represents a high-risk clinical scenario requiring rapid recognition and individualized management. This case highlights the importance of careful risk stratification, multidisciplinary assessment, and precise echocardiographic guidance. It also demonstrates that normotensive patients may exhibit early cardiogenic or obstructive shock physiology despite preserved systemic blood pressure. The complexity of this case lies in managing right-sided CIT and acute PE in the setting of severe biventricular dysfunction, requiring a multidisciplinary approach and the integration of complex anatomic, hemodynamic, and bleeding-risk considerations. In selected high-risk patients, percutaneous aspiration thrombectomy may offer a minimally invasive option for thrombus removal and hemodynamic improvement. This case also adds to the limited evidence supporting percutaneous aspiration thrombectomy as a potential intervention in normotensive patients with high-risk features and indicators of early shock physiology. Further research is needed to better define patient selection, procedural safety, and long-term outcomes in CIT.
